# Predicting poor postoperative acute pain outcome in adults: an international, multicentre database analysis of risk factors in 50,005 patients

**DOI:** 10.1097/PR9.0000000000000831

**Published:** 2020-07-27

**Authors:** Alexander Schnabel, Maryam Yahiaoui-Doktor, Winfried Meissner, Peter Konrad Zahn, Esther Miriam Pogatzki-Zahn

**Affiliations:** a Department of Anaesthesiology, Intensive Care and Pain Medicine, University Hospital Muenster, Muenster, Germany; b Institute for Medical Informatics, Statistics and Epidemiology, University of Leipzig, Leipzig, Germany; c Department of Anaesthesiology and Intensive Care, Jena University Hospital, Jena, Germany; d Department of Anaesthesiology and Intensive Care Medicine, Palliative Care Medicine and Pain Management, Berufsgenossenschaftliches Universitätsklinikum Bergmannsheil GmbH Bochum, Ruhr University Bochum, Germany

**Keywords:** Risk factors, Postoperative pain, Risk prediction, Database analysis, Chronification of pain

## Abstract

**Background:**

The aim of this study was to determine simple risk factors for severe pain intensity (≥7 points on a numeric rating scale [NRS]), to analyse their relation to other patient-reported outcome measures and to develop a simple prediction model.

**Methods:**

We used data from 50,005 patients from the PAIN-OUT project. Within a first data set (n = 33,667), relevant risk factors were identified by logistic binary regression analysis, assessed for additional patient-reported outcome measures beyond pain intensity and summed up for developing a simple risk score. Finally, sum of factors was plotted against postoperative pain outcomes within a validation data set (n = 16,338).

**Results:**

Odds ratios (OR) for the following risk factors were identified: younger age (<54 years, OR: 1.277), preoperative chronic pain at the site of surgery (OR: 1.195), female sex (OR: 1.433), duration of surgery (>90 minutes, OR: 1.308), preoperative opioid intake (OR: 1.250), feeling anxious (OR: 1.239) and feeling helpless due to pain (OR: 1.198), and the country of the recruiting centre (OR: 1.919). Patients with ≥3 risk factors had more severe pain intensity scores, spent a longer time in severe pain, and wished to have received more pain treatment (*P* < 0.001). A simple risk score was created with 4 risk factors showing a moderate prediction level.

**Conclusions:**

Patients with ≥3 risk factors are at higher risk for poor postoperative acute pain outcome after surgery. Future studies using this score might show that preventive strategies might decrease pain intensity, pain-related postoperative dysfunction, and the development of chronic pain.

## Abstract 2

Supplemental Digital Content is Available in the Text.

Eight risk factors were identified in a large international database. Patients with 3 or more risk factors are at higher risk for poor pain outcome.

## 1. Introduction

According to the results of several large surveys, postoperative pain is still undertreated and continues to be a relevant, unresolved problem worldwide.^3,7^ This is surprising because evidence-based guidelines for the treatment of acute postoperative pain are generated continuously.^1,5,21^ In addition, severe (and long-lasting) acute pain early after surgery is one major predictor for postoperative complications^26^ and chronic postoperative pain.^6^ One reason for the gap between available knowledge and insufficient therapy might be the different responses to pain and analgesics between individual patients.^8,11^ Thus, treatment strategies recommended in guidelines and for specific procedures might be sufficient for some but not all patients and optimising pain management might reach some but not all patients after surgery.^14,18^ This is even more important in the light of the opioid epidemic in several countries because high opioid prescriptions in patients with high pain scores failed to improve pain management.^10,23^

Just recently, a meta-analysis of 33 trials showed that there are 9 preoperative significant predictors for poor postoperative pain control.^28^ However, these results are limited because most of the included trials were unicentre, included retrospective data, and poor postoperative pain control was not defined a prori.

Therefore, the first aim of this study was to identify relevant risk factors for severe postoperative pain (defined as worst pain since surgery on numeric rating scale [NRS] ≥7 points^8^) after various surgical procedures by using the large multicentre international (26 countries, 76 centres) PAIN OUT data set.^30^ This database is based on exactly the same outcome questionnaire assessing a number of relevant patient-reported outcome parameters.^19^ In a second step, the odds ratio (OR) for each factor was assessed and the association with other patient-reported outcomes measures (PROMs) was examined. Finally, we determined a cutoff of the sum and calculated the specificity and sensitivity of this model to predict poor postoperative pain outcome.

## 2. Materials and methods

### 2.1. International postoperative pain outcome registry

Data collection was conducted within the PAIN OUT postoperative acute pain registry project, set up as part of a multinational and interdisciplinary project supported by the European Commission's Seventh Framework Program (FP7/2009–2013 under Grant Agreement No. 223590).^30^ The institutional review boards of all participating centres provided their approval for the project. Patients' Perception of Postoperative Pain Management was evaluated using the validated international pain outcome (IPO) questionnaire,^19^ which was translated into 8 languages and used according to the standardised procedure developed within the Pain Out project.^30^ The IPO covers 5 aspects (domains) of outcome measurement in acute pain, namely: pain severity (3 questions), functional interference of pain (4 questions), affective experiences related to pain, analgesic side effects, and the perception of care. In addition, patient characteristics, demographic, and clinical data comprising age, sex, comorbidities, type of surgery, and anaesthesia as well as analgesics administered in the perioperative period were captured. Each clinic recruited an average of 526 patients (range: 61–2261). Each participating centre had one or more study assistants trained and tested in data collection in both its paper and online forms. The assistants received a daily list of patients having had surgery on the previous day from the ward and randomly chose a number of them to recruit for the study. The patients who were eligible and consented to participate were given a paper version of the IPO, asked to fill it out, and hand it back to the assistants. The assistants later input these data into the online mask for the PAIN OUT registry, where the patient data were stored anonymously.

### 2.2. Patients

Patients aged of 18 or older, who were available (on the defined wards), had been operated the previous day, had spent a minimum of 6 hours on the ward, were not cognitively impaired, and had given their consent could be included in the PAIN OUT register. All patients recruited in 76 hospitals from 26 countries between January 2011 and July 2015 were included in the analysis for this study.

### 2.3. Data

Data from 50,005 in-patients were available for this study. Some of the patients and patient data were included in a recent analysis on patients' satisfaction after surgery.^17^ The data variables of interest for the present analysis fell into the following categories:

1. Patient characteristics (sex, age, body mass index, intensity of preoperative chronic pain [rated on a NRS; 0 = no pain—10 = maximum pain], and location of preoperative persistent pain [site of surgery and/or elsewhere, or elsewhere only]).
2. Perioperative patient care (consumption of preoperative opioids [yes/no])
3. Surgery parameters (duration of surgery [dichotomised based on the median value from our data set of 90 minutes], and country of the recruiting centre [dichotomised in countries with a population with a median worst pain NRS score <7 vs ≥7 points]).
4. Questions from the IPO questionnaire, which asked patients on an NRS scale of 0 (=not anxious, not helpless) to 10 (=maximum anxious, maximum helpless) whether they felt anxious due to pain and whether they felt helpless due to pain (2 separate questions).
5. Patient outcomes included worst pain intensity since surgery (rated on NRS [0 = no pain—10 = maximum pain]), percentage of time spent in severe pain (rated on NRS [0%–100% in 10% intervals]), wish for more treatment ([yes/no]), pain-related interference with activities in bed and respectively out of bed (rated on NRS [0 = no interference—10 = maximum interference]).

### 2.4. Statistical analyses

All statistical analyses were conducted using R version 3.4.2 (R Core Team, 2016. R: A language and environment for statistical computing. R Foundation for Statistical Computing, Vienna, Austria. URL https://www.r-project.org). For all analyses, an alpha level of 0.05 was used to determine statistical significance (2-tailed). Severe postoperative pain was defined as worst pain since surgery ≥7 NRS points based on a recently published analysis focusing on risk factors for severe postoperative pain in more than 22,000 German patients from another project.^8^ We randomly divided our data into a test (2/3) and a validation data set (1/3).

First, we ran a logistic binary regression on the dichotomised item severe postoperative pain (yes/no) within the test population and calculated the ORs together with their 95% confidence interval (CI) to determine the most important risk factors influencing this outcome.

After an exploratory factor analysis, we summed up the number of risk factors (each factor was rated as 1) of each patient within the validation cohort. First, we plotted a receiver operating characteristic curve to determine the predictive goodness of the sum of all risk factors in determining the risk of having severe postoperative pain.

In addition, the patients' sum of risk factors were assessed for 3 postoperative pain-related outcomes (number of patients with worst pain since surgery ≥7 NRS points [vs <7 points]), patients with a 20% and more percentage of time spent in severe pain within the past 24 hours [vs patients with less than 20%], and number of patients wished to have received more pain treatment [vs the number of patients wished to have received no more pain treatment]). We rated the outcome of spending more than 20% of time in severe pain to be undesirable because this was the median with the whole validation data set. Furthermore, based on these analyses, we determined a possible relevant cutoff of the sum of risk factors, which might distinguish between patients at high and low risk for poor postoperative pain outcome and calculated the specificity and sensitivity. Apart from that, we compared severe postoperative pain with pain-related interference with activities in bed and out of bed. Finally, we created a reduced risk score by including only those 4 risk factors that are easy to assess and can therefore be used without any psychological preoperative assessment tools (“simple risk score”).

## 3. Results

### 3.1. Demographic data

A total of 50,005 data sets from patients included in the PAIN OUT registry (recruited between January 2011 and July 2015) were used for this study. Table 1 illustrates patient characteristics in the test (n = 33,667) and the validation (n = 16,338) data sets, and there were no significant differences between both groups. In the whole population, there were significant differences of the number of patients suffering from severe postoperative pain (≥7 NRS points) between the countries of the recruiting centres.

**Table 1:**
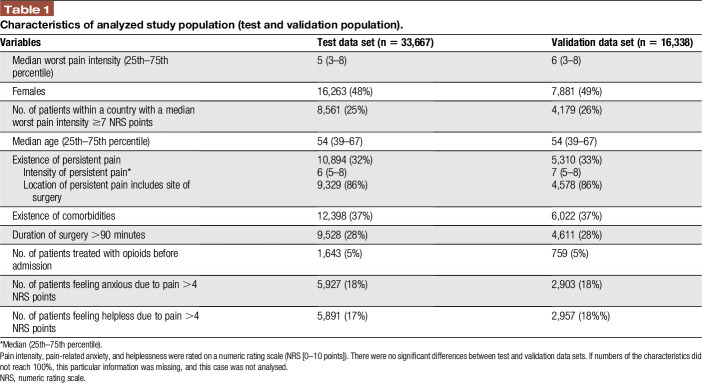
Characteristics of analyzed study population (test and validation population).

### 3.2. Identification of risk factors for severe postoperative pain in the test data set

Results from the binary logistic regression analysis (Table 2) within the test data set showed that female sex, younger age (lower than the median of 54 years within the test population), a country with a median worst pain intensity since surgery ≥7 NRS points, location of preoperative persistent pain (at the site of surgery and elsewhere or at the site of surgery only compared to preoperative persistent pain elsewhere alone), a preoperative opioid intake (vs no intake), a longer duration of surgery (longer than the median of 90 minutes within the test population), and a higher level of anxiety or helplessness (higher NRS score than the median of 4, which represented the 75% percentile within the test population) were the most relevant risk factors for severe postoperative pain at the first day after surgery. The most important risk factor for severe postoperative pain was the country where the surgery took place, with an OR of 1.916 (*P* < 0.001).

**Table 2:**
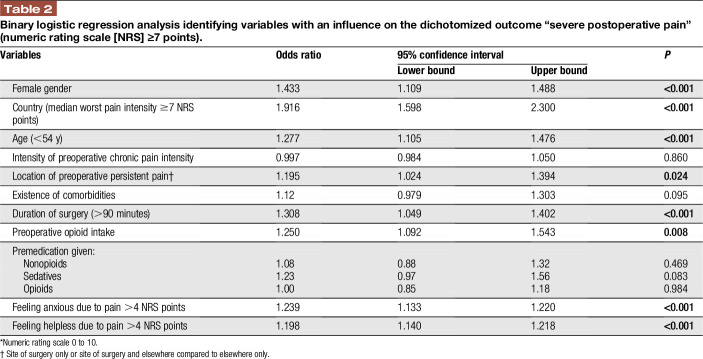
Binary logistic regression analysis identifying variables with an influence on the dichotomized outcome “severe postoperative pain” (numeric rating scale [NRS] ≥7 points).

### 3.3. Development of a prediction model

Based on the binary logistic regression analysis and after an exploratory factor analysis (see table, Supplemental Digital Content 1, results of the exploratory factor analysis, available at http://links.lww.com/PR9/A66) each factor, that significantly predicted severe pain, was rated as 1 and added up to obtain a sum (from 0 to 8) for the individual's risk of developing severe pain.

**Figure 1.**
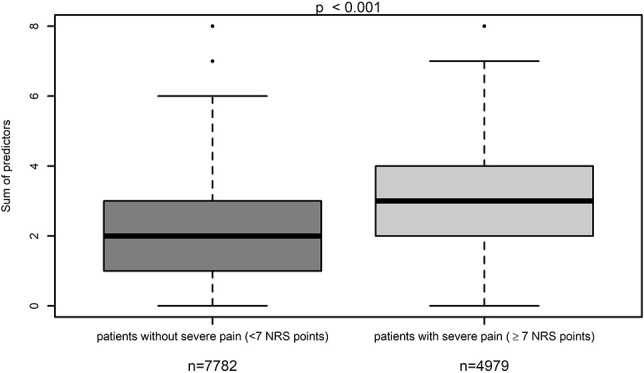
Comparison of the number of risk factors in patients suffering from pain scores < or ≥7 points on a numeric rating scale (NRS).

**Figure 2.**
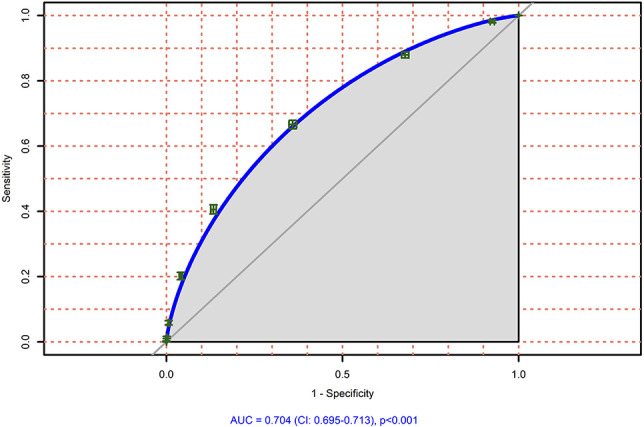
Area under the curve for the prediction of patients (original risk factors) within the validation cohort (n = 16338) suffering from severe postoperative pain.

**Figure 3.**
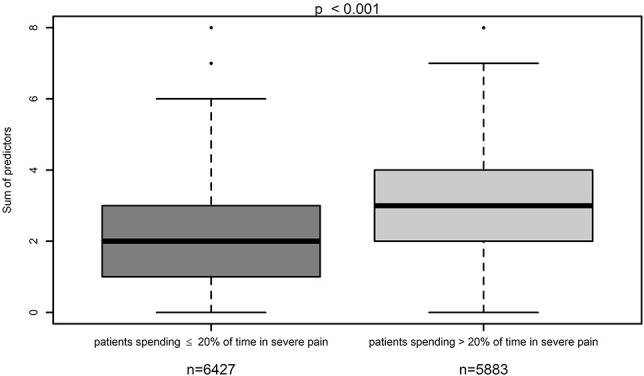
Comparison of the number of risk factors within the group of patients suffering from more than the 20% of time spending in severe pain in the last 24 hours.

**Figure 4.**
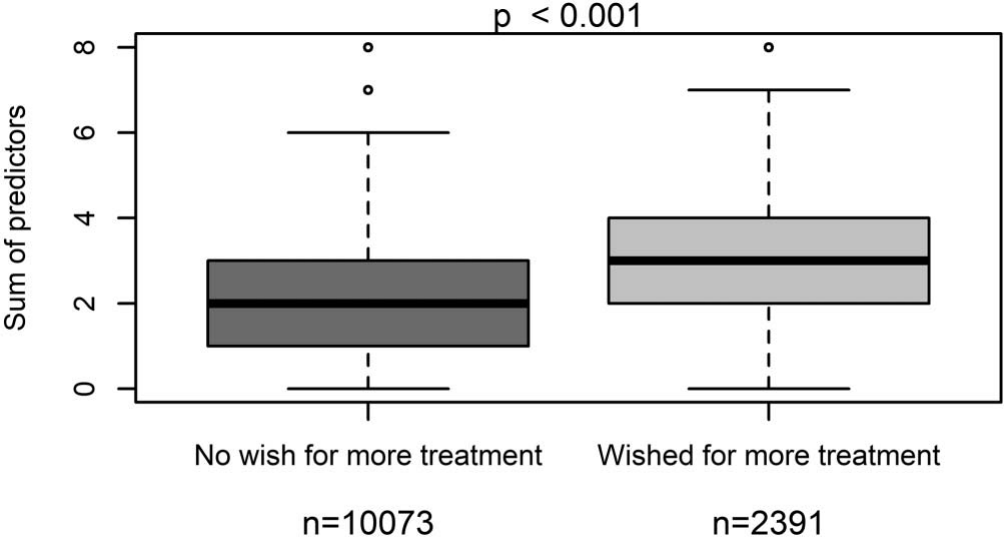
Comparison of the number of risk factors within the group of patients wishing to have received more pain treatment or not.

Within the validation cohort, we showed that patients with a median of 3 or more risk factors were significantly more often within the group of patients with severe postoperative pain, whereas patients with a median of less than 3 risk factors were significantly more often within the group of patients with lower worst pain intensities (NRS <7 points) (*P* < 0.001) (Fig. 1). We performed an ROC curve, which shows the predictive goodness of this model. An area under the curve (AUC) of 0.704 (95% CI: 0.695–0.713 *P* < 0.001) revealed a moderate level of prediction (Fig. 2). In addition, we demonstrated that patients with severe postoperative pain reported a higher interference of pain with activities in bed (see Figure, Supplemental Digital Content 3, comparison of maximum pain intensity with interference of pain with activities in bed, available at http://links.lww.com/PR9/A66), and similarly for activities out of bed (see Figure, Supplemental Digital Content 4, comparison of maximum pain intensity with interference of pain with activities out of bed, available at http://links.lww.com/PR9/A66). For interference of pain with activities in bed and activities out of bed, the Pearson correlation coefficient with existence of severe pain was 0.480 and 0.463, respectively. Furthermore, we demonstrated that patients with a median of 3 or more risk factors were significantly more often within the group of patients suffering from more than the 20% of time spending in severe pain, which represents the median within the whole validation data set (Fig. 3). In addition, we revealed that significantly more patients wished to have received more pain treatment, if they had a median of 3 or more risk factors (*P* < 0.001) (Fig. 4). According to these analyses, we concluded that the occurrence of 3 or more risk factors might distinguish between high-risk and low-risk patients for poor postoperative pain outcome related to different PROM. The percentage of patients with 3 or more risk factors within the test, respectively validation data set was comparable (37.9 or 37.8%). The calculated sensitivity and specificity for this cut off was 0.666 and 0.641, respectively, which represents again a moderate predictive value of this model.

Finally, we created a condensed risk score by including only those 4 preoperative risk factors (age, preoperative chronic pain, female sex, and preoperative opioid intake; see table, Supplemental Digital Content 2, description of the “simple risk score,” available at http://links.lww.com/PR9/A66) that are easy to assess and for which specific (and usually extensive) psychological assessment tools are not required. This “simplified risk score” showed a slightly lower but still moderate prediction level (AUC: 0.607, 95% CI: 0.601–0.613, *P* < 0.001) for poor postoperative pain outcome (NRS ≥7 points, time in severe pain and wish for more pain treatment, see Figures, Supplemental Digital Content 5, AUC for the prediction of severe postoperative pain; Supplemental Digital Content 6; relative frequencies of severe postoperative pain intensity in relation to the individual risk score; Supplemental Digital Content 7, frequency of wish for more pain treatment in relation to the individual risk score, available at http://links.lww.com/PR9/A66).

## 4. Discussion

The present multicentre international prospective database analysis including 50,005 data sets from 26 countries and 76 hospitals demonstrates that younger age, female sex, preoperative opioid intake, preoperative persistent pain at the site of surgery and elsewhere or at the site of surgery alone, longer duration of surgery, higher pain-related anxiety, and/or helplessness are major risk factors for suffering from severe postoperative pain (NRS ≥7 points); in addition, the country, where the patient was operated, showed different risk estimates. Patients with 3 or more risk factors had an increased risk for severe pain after surgery. The risk for other pain-related outcome parameters (longer time in severe pain and wish for more pain treatment) increased as well by summing up the risk factors.

### 4.1. Risk factors for severe postoperative pain

Our multicentre international data analysis confirmed some risk factors for severe postoperative pain identified in an analysis of the German acute pain registry^8^ (female sex and young age) and the results of a recently published meta-analysis focusing on preoperative predictors for poor postoperative pain control (female sex, young age, preoperative analgesia, and pain-related anxiety symptoms).^28^ However, some risk factors suggested earlier were not confirmed by us (such as “body mass index”) and some others were added to those suggested earlier: country, preoperative opioid use, preoperative persistent pain at the site of surgery and elsewhere or at the site of surgery only, a longer duration of surgery, pain-related helplessness, and anxiety.

In our international database, the predictor “country” “was one of the most relevant risk factors for severe postoperative pain. As shown recently, mean pain intensity ratings of patients after orthopedic surgery differed between industrial countries.^4,29^ The difference in the present database analysis might therefore not only reflect quality differences in pain treatment or different preoperative risk profiles for high pain intensities within the populations (eg, more patients with preoperative opioids), but also different cultural aspects and pain expectations within the patients experiencing postoperative pain. The other risk factors such as “preoperative opioid use,” “preoperative persistent pain at the site of surgery and elsewhere” or “preoperative pain at the site of surgery only,” and “longer duration of surgery” clearly indicate that a higher preoperative and intraoperative sensitization of the nociceptive system might be predictive for severe postoperative pain and poor postoperative pain control.^9^ In addition, a published longitudinal trial focusing on the development of chronic neuropathic postoperative pain after breast cancer surgery showed that preoperative pain in the surgical area and other chronic pain states were associated with chronic neuropathic pain.^16^ Thus, preoperative pain sensitization due to chronic pain and opioid consumption before surgery might be relevant for both, acute and chronic pain after surgery and might be connected somehow to each other. This is supported by the fact that “duration of severe pain after surgery,” one outcome tested here, is predictive of chronic pain after surgery.^6^

There is ample evidence that psychological factors are important predictors for postoperative pain.^11,22,28^ We also included 2 psychological factors; due to study design properties, we were only able to include data from the first postoperative day.^30^ However, because anxiety levels seem to remain stable perioperatively,^15^ we most likely assessed a trait than a state phenomenon. Chronic postoperative pain might be predicted by anxiety as well, although catastrophizing might be more relevant.^24^ However, the role of preoperative psychological aspects for acute postoperative pain needs further investigations.^20^

### 4.2. Development of a clinically relevant and easy-to-use predictive risk score

Besides risk assessment we show here, the predictive factors seem to be—at least in part—independent from each other. We therefore developed a prediction model by summing up the risk factors for severe postoperative pain intensity. Our data indicate that patients with 3 or more risk factors are not only at higher risk for severe postoperative pain intensity but also for 2 other PROMs (“time in severe pain” and “wish to receive more pain treatment”). The validation analysis showed a moderate predictive value with an AUC of 0.704 (95% CI: 0.695–0.713 *P* < 0.001). Thus, the model seems to be acceptable predictive for several pain-related outcome parameters, which might be also relevant for chronic pain after surgery.^6^ Apart from pain intensity, we also showed that severe postoperative pain resulted in higher interference of pain with activities in bed and out of bed. This indicates our score is able to predict not only pain intensity but also pain-related impairment of physical function. However, because impairment of physical function due to pain was not the primary outcome of our study, this should be investigated in the future. One disadvantage of the PAIN OUT data set is that PROMs are assessed at only one single time point (eg, the first day after surgery). As a consequence, we do not have preoperative assessment tools for the 2 psychological risk factors that turned out to be significant in our analysis (anxiety and helplessness). We therefore created a condensed “simple risk score” by including 4 preoperatively available (and easy-to-assess) risk factors (age, preoperative chronic pain, female sex, and preoperative opioid intake, see Table 3) that can be used without any psychological preoperative assessment. This risk score showed still an acceptable predictability for poor postoperative pain outcome. The predictability might be increased by including psychological factors, but exact assessment tools and cutoffs need to be determined (or even developed).

**Table 3:**
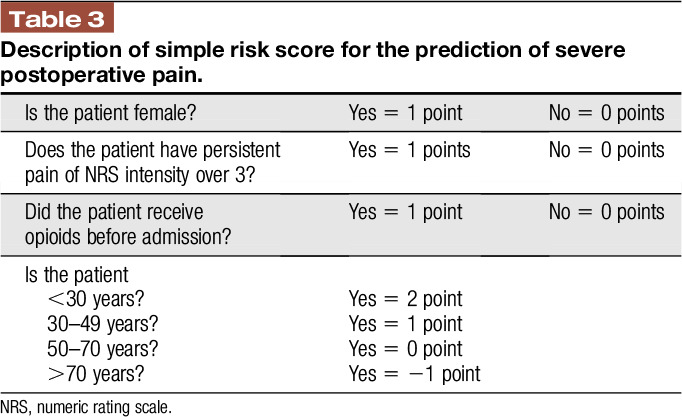
Description of simple risk score for the prediction of severe postoperative pain.

There are currently 2 other risk scores available for the prediction of severe pain after surgery.^12,13,17^ Both scores^12,13,17^ showed comparable prediction levels compared to ours; however, they were neither confirmed in international data sets nor in patients with different surgical procedures. In addition, the calculation method for both is more difficult in the daily clinical setting because they use a distinct calculation program.

### 4.3. Potential benefits of the predictive risk score

Although some risk scores exist (see above), their use for predicting patients with poor postoperative pain seem to be rare possibly because their use is too difficult in daily clinical practice, therefore the need for a simple risk score remains. The recently published multicentre approach was able to improve acute postoperative pain management in 6 hospitals of one German city by using guideline-related pain assessment and treatment strategies.^18^ Although it was successful, pain in patients with preoperative chronic pain (one of the risk factors incorporated here) did not improve, indicating that a risk-related treatment might be required. Another recently published observational trial demonstrated that a more intense perioperative pain treatment in women with a high risk for severe postoperative pain identified preoperatively by using the Pain score^17^ significantly improved pain outcome after caesarean section.^2^ Several observational reports showed that a perioperative multimodal pain treatment by a transitional pain service improved postoperative care in patients at a high risk for severe postoperative pain and pain chronification.^10,25,27^ Our risk score might be a useful tool to identify patients with a need for such interdisciplinary approaches.

### 4.4. Limitations

The use of PAIN OUT data enabled us to include a large number of patient data prospectively collected by using a validated outcome questionnaire in 76 hospitals across 26 countries. One limitation is, however, the restriction of the Pain Out project sample to the first day after surgery only. However, day one is usually the day with the most intense pain scores after surgery. In addition, previous studies indicated that exactly this time point is related to the development of chronic pain after surgery.^6^

Another limitation of such a large international database might be that postoperative pain treatment was not standardised. The advantage of such a strategy is that the factors are derived from observational, everyday clinical data and thus are transferable to hospitals irrespective of their pain management strategies all over the world. The disadvantage might be that the risk differs related to the treatment.

The PAIN OUT data set collects a limited amount of data from each patient, eg, because preoperative data are not directly assessed or pain intensity at rest or during movement was not evaluated specifically. However, this enables the project to collect data from a high number of patients. In addition, the use of a validated patient-reported outcome questionnaire to collect data gathers valid data and enables direct comparison between hospitals and countries.

### 4.5. Conclusion

In conclusion, our analysis based on a large data set from an international acute pain registry demonstrates valid OR for risk factors related to poor pain-related outcome at the first day after surgery. The development of an easy-to-use predictive risk model (based on the sum of these risk factors) with a moderate prediction level showed that the proportion of patients with severe pain and other postoperative pain outcomes increased significantly with 3 or more risk factors.

## Disclosures

The authors have no conflicts of interest to declare.

This work was supported by the European Commission's Seventh Framework Program (FP7/2009-2013 under Grant Agreement No. 223590) to E.M. Pogatzki-Zahn and to W. Meissner (coordinator).

## Appendix A. Supplemental digital content

Supplemental digital content associated with this article can be found online at http://links.lww.com/PR9/A66.

## Supplementary Material

SUPPLEMENTARY MATERIAL
