# Supplementary material for: Predicting poor postoperative acute pain outcome in adults: an international, multicentre database analysis of risk factors in 50,005 patients
Source: Pain Rep. 2020 Jul 27;5(4):e831. doi: 10.1097/PR9.0000000000000831 (PMC7390596; doi:10.1097/PR9.0000000000000831)
Supplement: SUPPLEMENTARY MATERIAL [file painreports-5-e831-s001.docx]

Table Supplemental Digital Content 1 The components from the principal component analysis of the constituent factors of the sum

|  | Factor 1 | Factor 2 | Factor 3 | Factor 4 |
| --- | --- | --- | --- | --- |
| Gender | 0.201 | 0.161 |  | ***-0.537*** |
| Feeling anxious due to pain | ***0.862*** |  |  |  |
| Feeling helpless due to pain | ***0.864*** |  |  |  |
| Duration of surgery | 0.140 | ***-0.702*** |  |  |
| Location of persistent pain | 0.122 |  |  | ***0.849*** |
| Country |  | 0.104 | ***0.728*** | -0.174 |
| Age |  | ***0.750*** |  |  |
| Received opioids prior to admission |  | -0.120 | ***0.754*** | 0.160 |
