# Supplementary material for: Predicting poor postoperative acute pain outcome in adults: an international, multicentre database analysis of risk factors in 50,005 patients
Source: Pain Rep. 2020 Jul 27;5(4):e831. doi: 10.1097/PR9.0000000000000831 (PMC7390596; doi:10.1097/PR9.0000000000000831)
Supplement: SUPPLEMENTARY MATERIAL [file painreports-5-e831-s002.docx]

Table Supplemental Digital Content 2 Description of simple risk score for the prediction of severe postoperative pain.

| **Is the patient female?** | Yes = 1 point | No = 0 points |
| --- | --- | --- |
| **Does the patient have persistent pain of NRS intensity over 3?** | Yes = 1 points | No = 0 points |
| **Did the patient receive opioids before admission?** | Yes = 1 point | No = 0 points |
| **Is the patient**  **< 30 years?**  **30 – 49 years?**  **50 – 70 years?**  **> 70 years?** | Yes = 2 point  Yes = 1 point  Yes = 0 point  Yes = -1 point | |
