# Supplementary figures and images for: Predicting poor postoperative acute pain outcome in adults: an international, multicentre database analysis of risk factors in 50,005 patients
Source: Pain Rep. 2020 Jul 27;5(4):e831. doi: 10.1097/PR9.0000000000000831 (PMC7390596; doi:10.1097/PR9.0000000000000831)

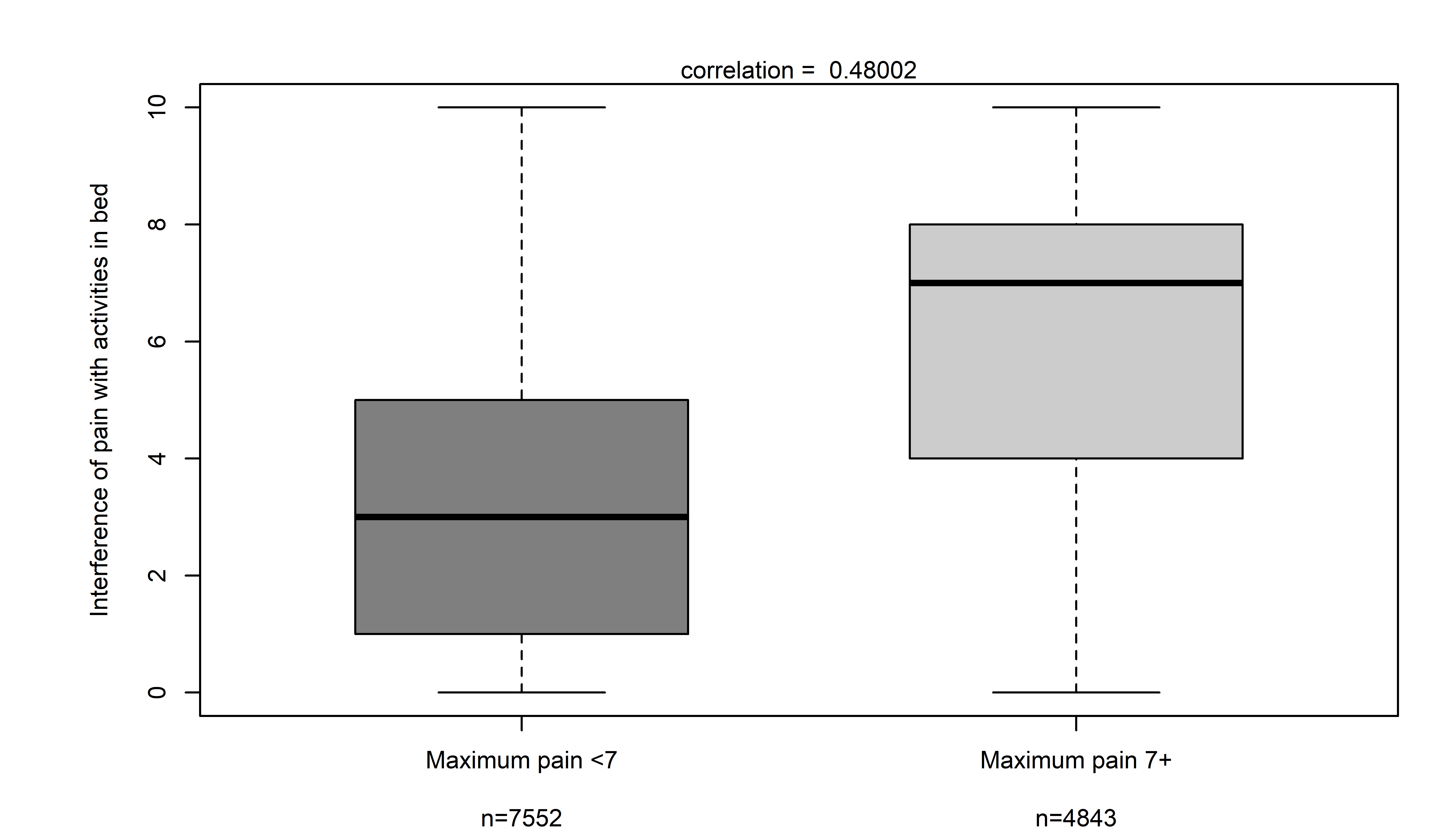

Supplement: SUPPLEMENTARY MATERIAL [file painreports-5-e831-s003.jpg]

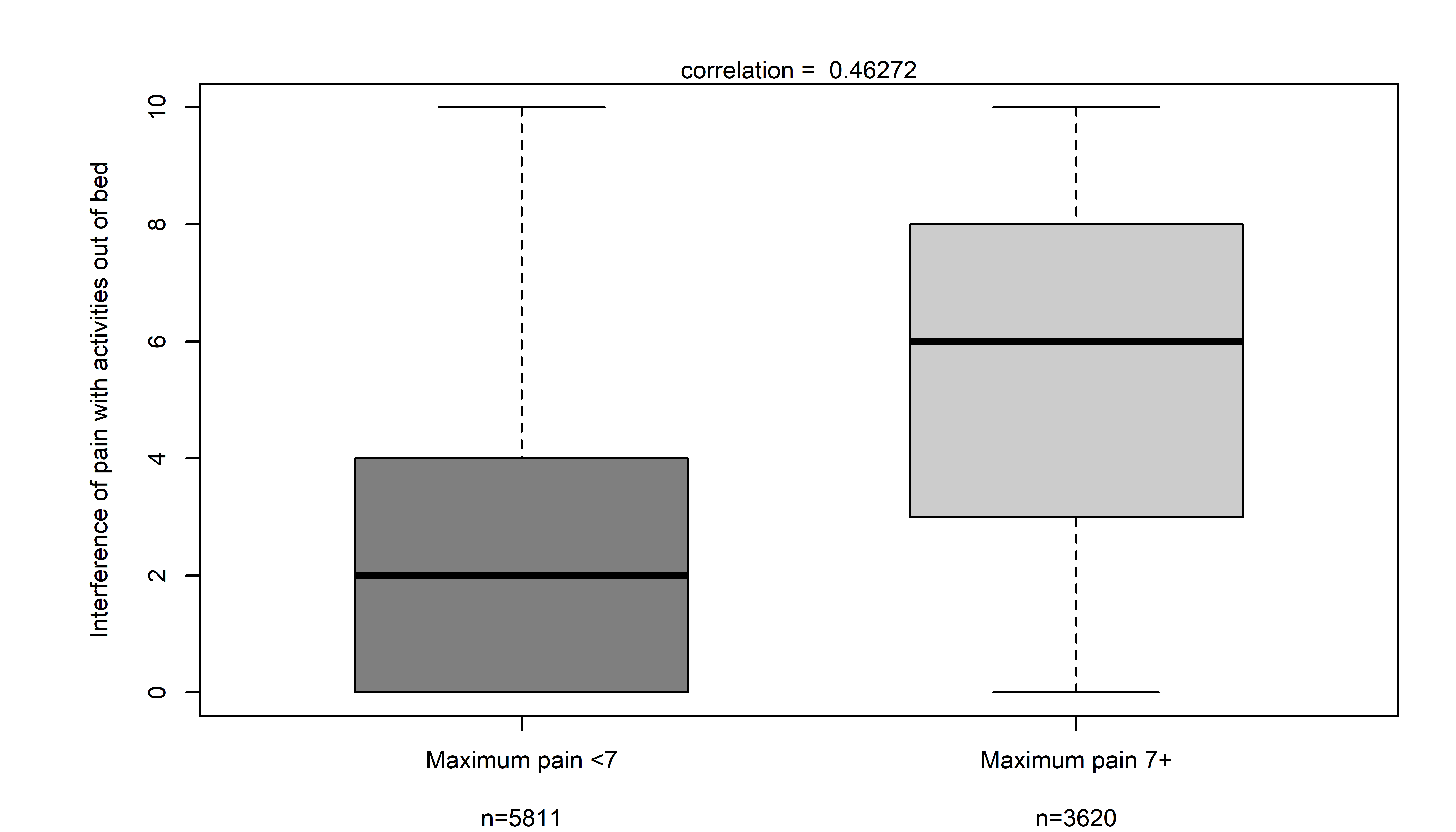

Supplement: SUPPLEMENTARY MATERIAL [file painreports-5-e831-s004.jpg]

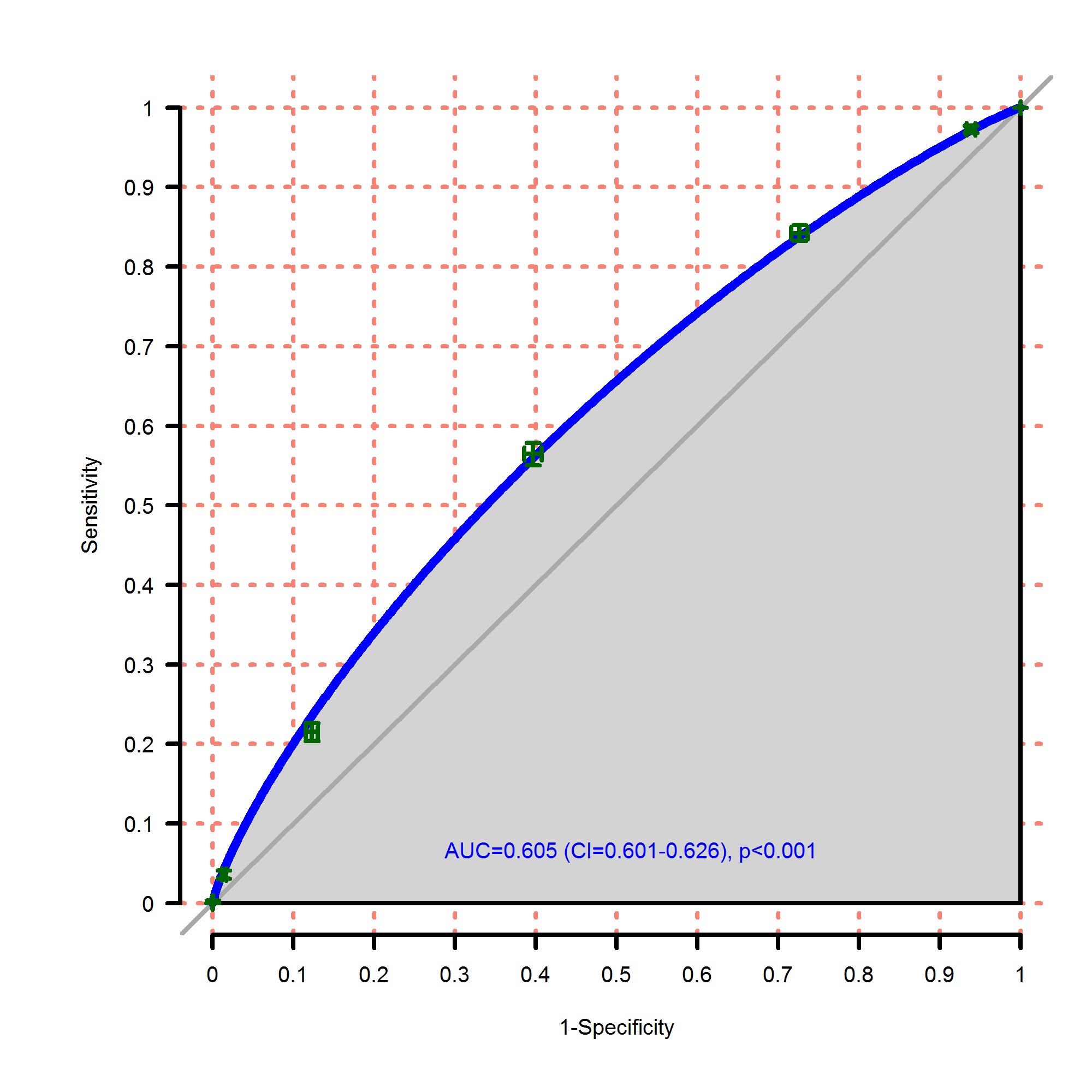

Supplement: SUPPLEMENTARY MATERIAL [file painreports-5-e831-s005.jpg]

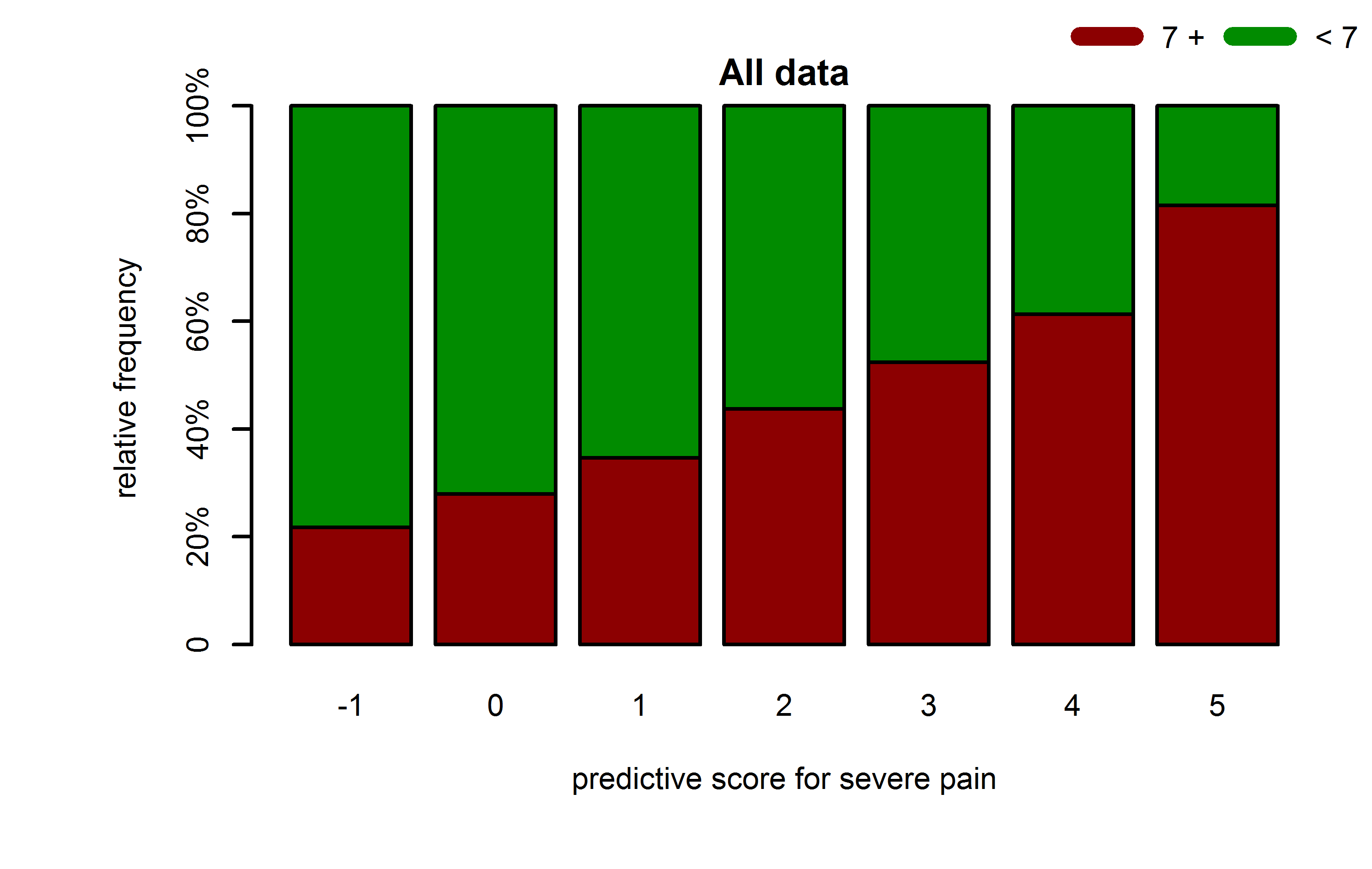

Supplement: SUPPLEMENTARY MATERIAL [file painreports-5-e831-s006.jpg]

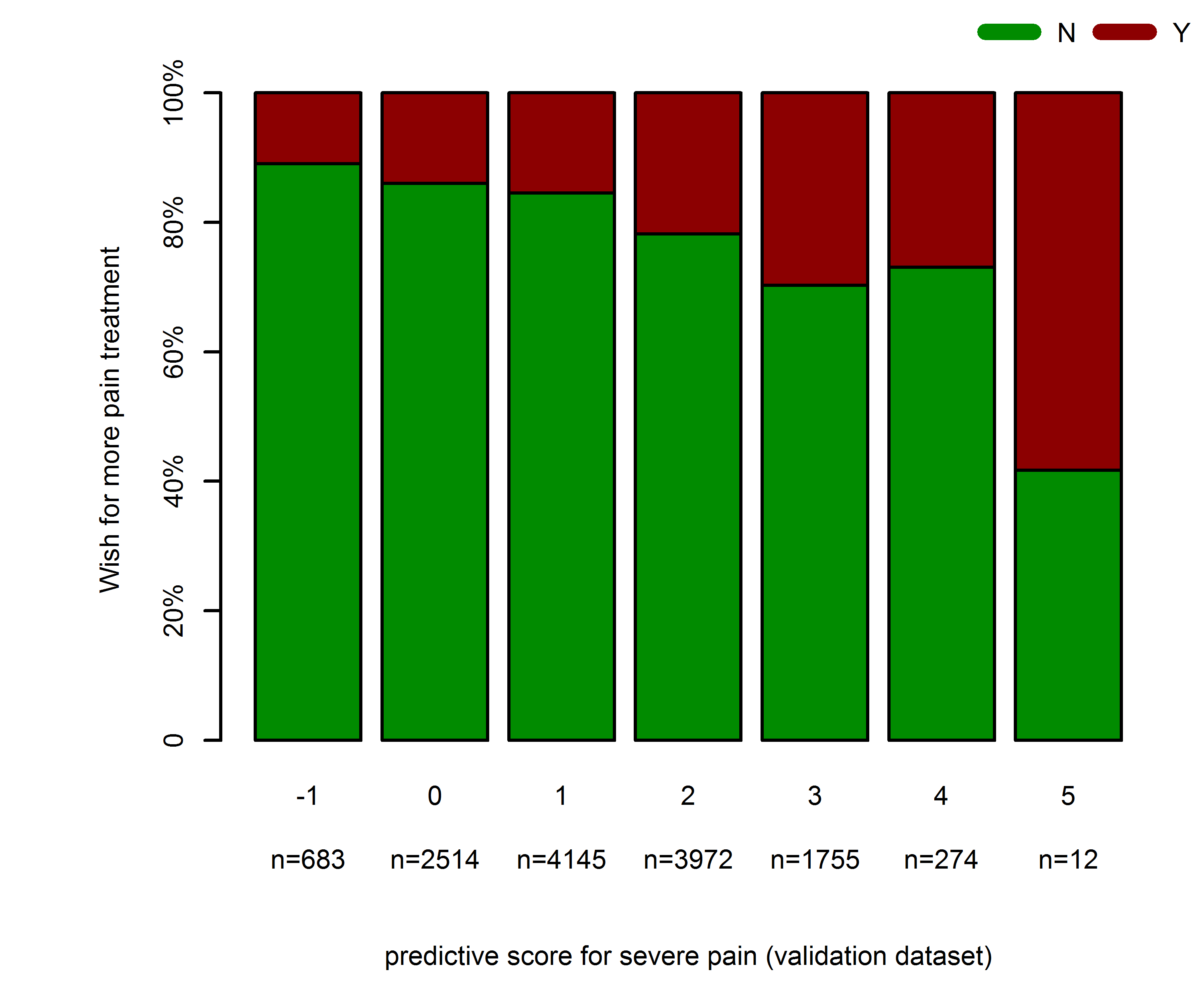

Supplement: SUPPLEMENTARY MATERIAL [file painreports-5-e831-s007.jpg]
